# Left Atrial Appendage Occlusion in Patients With Atrial Fibrillation and Cancer

**DOI:** 10.1016/j.jaccao.2022.10.016

**Published:** 2023-03-07

**Authors:** Samuel A. Shabtaie, Nicholas Y. Tan, Robert C. Ward, Bradley R. Lewis, Eric H. Yang, David R. Holmes, Joerg Herrmann

**Affiliations:** a Department of Cardiovascular Medicine, Mayo Clinic, Rochester, Minnesota, USA; b Department of Biostatistics, Mayo Clinic, Rochester, Minnesota, USA; c Department of Cardiovascular Medicine, Mayo Clinic, Phoenix, Arizona, USA

**Keywords:** atrial fibrillation, cancer, left atrial appendage occlusion, malignancy, stroke prevention, Watchman

## Abstract

**Background:**

The prevention of stroke in patients with atrial fibrillation (AF) and cancer is challenging because patients are at increased bleeding and thrombotic risk.

**Objectives:**

The authors sought to assess left atrial appendage occlusion (LAAO) as a safe and effective strategy for reducing stroke at no increased bleeding risk in cancer patients with AF.

**Methods:**

We reviewed patients with nonvalvular AF who underwent LAAO at Mayo Clinic sites from 2017 to 2020 and identified those who had undergone prior or current treatment for cancer. We compared the incidence of stroke, bleeding, device complications, and death with a control group who underwent LAAO without malignancy.

**Results:**

Fifty-five patients were included; 44 (80.0%) were male, and the mean age was 79.0 ± 6.1 years. The median CHA_2_Ds_2_-VASc score was 5 (Q1-Q3: 4-6), with 47 (85.5%) having a prior bleeding event. Over the first year, ischemic stroke occurred in 1 (1.4%) patient, bleeding complications in 5 (10.7%) patients, and death in 3 (6.5%) patients. Compared with controls who underwent LAAO without cancer, there was no significant difference in ischemic stroke (HR: 0.44; 95% CI: 0.10-1.97; *P =* 0.28), bleeding complication (HR: 0.71; 95% CI: 0.28-1.86; *P =* 0.19), or death (HR: 1.39; 95% CI: 0.73-2.64; *P =* 0.32).

**Conclusions:**

Within our cohort, LAAO in cancer patients was achieved with good procedural success and offered a reduction in stroke at no increased bleeding risk similar to noncancer patients.

Atrial fibrillation (AF) poses a risk of ischemic stroke that is commonly attempted to be mitigated by oral anticoagulation.^1^^,^^2^ This increased risk is theorized to stem from clot formation within the left atrial appendage.^3^^,^^4^ In patients with nonvalvular AF for whom oral anticoagulation is contraindicated or not felt to be desirable, left atrial appendage occlusion (LAAO) with a Watchman device (Boston Scientific) is an alternative option for stroke risk reduction.^5^, ^6^, ^7^, ^8^

It is well established that patients with malignancies have an increased risk of clot formation and thromboembolism.^9^^,^^10^ Furthermore, it has been shown that patients with cancer are at increased risk of ischemic stroke compared with the general population.^11^ However, among patients with cancer, the cause of ischemic stroke remains uncertain in up to 50% of cases.^12^ Additionally, cancer patients on oral anticoagulation have an increased risk of bleeding compared with those without any malignancy,^13^ complicating the management of patients with concomitant AF and cancer. Patients with cancer are surviving longer, and the incidence of AF continues to rise as the general population ages.^14^^,^^15^ This poses the challenge to the clinician of how best to manage the risks associated with both AF and cancer.

There is currently a paucity of data regarding the safety and efficacy of LAAO in patients with AF and malignancy. We sought to retrospectively review all patients with malignancy who underwent LAAO at Mayo Clinic. We hypothesized that LAAO would be efficacious in preventing stroke in patients with cancer similar to patients without cancer.

## Methods

We retrospectively reviewed all patients who underwent LAAO at 1 of the Mayo Clinic sites between January 1, 2017, and November 1, 2020, and identified those who had active or prior malignancy. To be eligible for study participation, subjects must have been at least 18 years of age at the time of LAAO and provided research consent. From this group, we obtained demographic and laboratory data as close as possible to the date of LAAO in addition to the last clinical follow-up. We used an institutional cancer registry to obtain information regarding malignancy history (active vs remission), cancer type/stage, and treatments rendered. Active cancer was defined as the patient receiving cancer-directed treatments (chemotherapy, surgery, or hormonal therapy) within 6 months of device implantation or those with untreated disease. We then obtained information regarding AF history including duration; CHA_2_Ds_2_-VASc score; HAS-Bled score; cardiovascular risk factors; left ventricular ejection fraction; medications; and treatments obtained via the National Cardiovascular Data Registry. Given the comprehensive parameters and follow-up of the National Cardiovascular Data Registry, this was used to assess baseline characteristics for all study participants. Primary outcomes included the incidence of stroke (ischemic and hemorrhagic) or transient ischemia attack, a major adverse cardiac event, major bleeding, or death. Secondary outcomes included acute procedural success and periprocedural/device complications. Procedural success was defined as efficacious LAAO device deployment without unintentional migration. A device perileak was only reported if it was >5 mm on transesophageal imaging upon follow-up. We then compared this cohort with all patients who underwent LAAO at any of the Mayo Clinic sites within the same time frame with no cancer history. We evaluated the stroke incidence, bleeding incidence, death (any cause), and periprocedural/device complications between the 2 groups. This study was approved by the Institutional Review Board of the Mayo Clinic.

### Statistical analysis

Continuous variables were reported as mean ± SD or median and 25th to 75th percentile range (quartile Q1-Q3) unless otherwise indicated. Categoric variables were reported as frequency (percentage). We established a control group of patients who underwent LAAO device implantation at our institution during the same time frame who did not have a history of active or past malignancy. Inverse probability weights were calculated to adjust for the effects of age and sex between cancers and controls. This was accomplished by fitting a logistic regression with age and sex as independent variables and cancer status as the dependent variable. The probability of being in either group was then calculated for each patient. The subject’s weight was the inverse of the probability of being in the group in which the patient was a member.^16^ Balance was assessed using absolute standardized differences. Both age and sex were found to be below 0.1, indicating good balance. Long-term outcomes were modeled using Cox proportional hazards regression, and cumulative incidence curves were made. In the presence of the competing risk of death (or the alternative stroke or device event), marginal event rates were used in cumulative incidence curves. Because the goals of this study were etiologic in nature, cause-specific Cox proportional hazards models were the primary statistical test.^17^ Additionally, Fine and Gray subdistribution HRs and *P* values were included for comparison. Survival models are presented as HRs with 95% CIs. All outcomes other than mortality were analyzed from a competing risks framework. Schoenfeld residuals were used to assess the proportional hazards assumption, and no significant deviations from the assumption were detected. Percentages shown alongside event counts are cumulative incidence estimates. All *P* values are 2-sided, and *P* values <0.05 were considered significant for the purposes of this paper. All analyses were conducted using R version 4.0.3 (R Foundation for Statistical Computing).

## Results

A total of 55 patients (mean age 79.0 ± 6.1 years, 80% male) with malignancy and LAAO device implantation were included for analysis (Table 1). Of these, 52 (94.5%) had hypertension; 18 (32.7%) had heart failure; 17 (30.9%) had diabetes mellitus; 21 (38.2%) had suffered a previous stroke; 8 (14.5%) had suffered a previous transient ischemic attack; and 24 (43.6%) had vascular disease including previous myocardial infarction, peripheral arterial disease, or aortic atherosclerosis. Paroxysmal AF was present in 24 (43.6%) patients, persistent AF in 19 (34.5%), and permanent in 12 (21.8%). The median (Q1-Q3) CHA_2_Ds_2_-VASc score was 5 (Q1-Q3: 4-6), whereas the median HAS-Bled score was 3 (Q1-Q3: 2-4). A total of 47 (85.5%) patients had suffered a prior bleeding event with 21 (38.2%) intracranial events, 20 (36.4%) gastrointestinal events, 8 (14.5%) epistaxes, and 3 (5.5%) genitourinary bleeding events. Before device implantation, 49 (89.1%) were prescribed anticoagulation. The mean left atrial volume index by biplane volumetric measurement was 52.8 ± 21.7 mL/m^2^ (normal in our echocardiography laboratory is <34 mL/m^2^).

**Table 1:** Baseline Characteristics of Study Participants

	Cancer Patients	Control Group	*P* Value
Female	11 (20.0)	69 (32.5)	0.070
Male	44 (80.0)	143 (67.5)	0.070
Age, y	79.0 ± 6.1	76.8 ± 7.4	0.040
Implantation center			
Mayo Clinic–Rochester	31 (56.4)	137 (64.6)	0.26
Mayo Clinic–Arizona	24 (43.6)	75 (35.4)	0.26
Echocardiographic data			
Left ventricular ejection fraction, %	60 (53.5-64.5)	60 (55.0-65.0)	0.81
Laboratory studies			
Creatinine	1.2 (1.0-1.5)	1.3 (0.9-1.4)	0.51
Hemoglobin	13.2 (11.2-14.1)	12.6 (11.0-14.0)	0.70
Platelets	186,000 (135,000-262,500)	189,000 (153,000-224,750)	0.43
INR	1.1 (1.1-1.3)	1.2 (1.1-1.3)	0.61
CHA_2_DS_2_-VASc score	5 (4-6)	5 (4-6)	1.00
Heart failure	18 (32.7)	56 (26.4)	0.35
Hypertension	52 (94.5)	194 (91.5)	0.46
Diabetes	17 (30.9)	63 (29.7)	0.86
Stroke	21 (38.2)	73 (34.4)	0.79
Transient ischemic attack	8 (14.5)	26 (12.3)	0.65
Thromboembolism	18 (32.7)	64 (30.2)	0.14
Vascular disease	24 (43.6)	108 (50.9)	0.33
Age ≥75 y	39 (70.9)	140 (66.0)	0.49
HAS-BLED score	3 (2, 4)	3 (2, 4)	1.00
Hypertension (uncontrolled)	21 (38.2)	22 (10.4)	<0.001
Abnormal renal function	13 (23.6)	40 (18.9)	0.43
Abnormal liver function	4 (7.3)	8 (3.8)	0.26
Stroke	21 (38.2)	73 (34.4)	0.78
Bleeding	47 (85.5)	190 (89.6)	0.38
Labile INR	6 (10.9)	17 (8.0)	0.50
Alcohol	1 (1.8)	6 (2.8)	0.68
Antiplatelet medications	23 (41.8)	38 (17.9)	<0.001
NSAID use	3 (5.5)	12 (5.7)	0.95
Age >65 y	53 (96.4)	202 (95.3)	0.73
Classification of atrial fibrillation			
Paroxysmal	24 (43.6)	97 (45.8)	0.78
Persistent	19 (34.5)	68 (32.1)	0.73
Permanent	12 (21.8)	47 (22.2)	0.51
Bleeding risk factors			
Increased fall risk	26 (47.3)	73 (34.4)	0.079
Prior bleeding event	47 (85.5)	191 (90.1)	0.32
Epistaxis	8 (14.5)	17 (8.0)	0.14
Gastrointestinal	20 (36.4)	87 (41.0)	0.53
Genitourinary	3 (5.5)	26 (12.3)	0.15
Intracranial	21 (38.2)	81 (38.2)	0.99
Concurrent anticoagulation	49 (89.1)	162 (76.4)	0.039
Clinical variables			
Prior cardiac surgery	9 (16.4)	40 (18.9)	0.67
Coronary artery bypass grafting	7 (12.7)	23 (10.8)	0.15
Valvular intervention	3 (5.5)	21 (9.9)	0.23
Presence of cardiomyopathy	9 (16.4)	34 (16.0)	0.95
Coronary artery disease	27 (49.1)	94 (44.3)	0.53
Obstructive sleep apnea	26 (47.3)	77 (36.3)	0.14
Chronic lung disease	10 (18.2)	30 (14.2)	0.46

Values are n (%), mean ± SD, or median (Q1-Q3).

INR = international normalized ratio; NSAID = nonsteroidal anti-inflammatory drug.

The mean age at cancer diagnosis was 70.7 ± 7.6 years. Twelve (21.8%) patients had active malignancy at the time of device implantation. Most malignancies were nonmetastatic (n = 42, 76.4%), with 40 (72.7%) patients having surgical intervention, 13 (23.6%) undergoing chemotherapy, 11 (20.0%) receiving radiation therapy, 7 (12.7%) receiving hormonal therapy, and 1 (1.8%) receiving immunotherapy. A full list of malignancy types is outlined in Table 2.

**Table 2:** Baseline Cancer-Related Variables of Study Participants (N = 55)

Age at cancer diagnosis, y	70.7 ± 7.6
Active at time of device implant	12 (21.8)
Remission at time of device implant	43 (78.2)
Localized	42 (76.4)
Metastatic	12 (21.8)
Treatment modalities	
Chemotherapy	13 (23.6)
Immunotherapy	1 (1.8)
Hormonal therapy	7 (12.7)
Radiation therapy	11 (20.0)
Prior surgery	40 (72.7)
Cancer type	
Genitourinary	
Bladder	3 (5.5)
Prostate	16 (29.1)
Renal	5 (9.1)
Gastrointestinal	
Colorectal	4 (7.3)
Esophageal	1 (1.8)
Liver	2 (3.6)
Pancreatic	1 (1.8)
Hematologic	
Acute myeloid leukemia	1 (1.8)
Diffuse large B-cell lymphoma	1 (1.8)
Multiple myeloma	2 (3.6)
Myelodysplastic syndrome	1 (1.8)
Polycythemia vera	1 (1.8)
Skin	
Melanoma	6 (10.9)
Merkel cell	1 (1.8)
Other	
Breast	4 (7.3)
Lung	2 (3.6)
Oropharyngeal	7 (12.7)
Thyroid	3 (5.5)
Metastatic carcinoid tumor	1 (1.8)
Schwannoma	1 (1.8)

Values are mean ± SD or n (%).

The indications for LAAO device implantation are outlined in Table 3, with 98.2% at increased risk of stroke and 89.1% with a history of major bleeding. All attempted implantations were successful with no periprocedural mortality. A total of 6 patients suffered complications within 30 days of the procedure including 4 (7.3%) patients with access site hematomas, 1 (1.8%) patient with pericarditis, and 1 (1.8%) patient with seizure (Table 4). None of the complications were life-threatening.

**Table 3:** Indication for Watchman Implantation Among Study Participants (N = 55)

Increased stroke risk	54 (98.2)
History of major bleeding	49 (89.1)
High fall risk	24 (43.6)
Labile INR	3 (5.5)
Patient preference	7 (12.7)
Noncompliance with anticoagulation	0 (0.0)

Values are n (%).

INR = international normalized ratio.

**Table 4:** Primary and Secondary Outcomes Among Study Participants (N = 55)

	N	1-Year Cumulative Incidence (%)	5-Year Cumulative Incidence (%)
Procedural complication within 30 days	6		
Vascular access site bleeding	4		
Pericarditis	1		
Seizure	1		
Myocardial infarction	0		
Stroke	3	2.9	4.9
Ischemic	2	1.4	3.4
Hemorrhagic	1	1.5	1.5
Device-related complication	6	5.6	8.3
Device thrombosis	4	4.2	7.2
Peridevice leak	2	1.1	3.3
Death	14	6.5	49.9
Unknown	3		
Cancer related	5		
Infection	4		
Other	2		
Bleeding complication	5	10.7	10.7
Gastrointestinal	2		
Genitourinary	1		
Epistaxis (requiring transfusion)	1		
Pelvic hemorrhage secondary to fall	1		

The type and duration of antithrombotic therapy after implantation was at the discretion of the implanting physician. Most patients were discharged on an antithrombotic regimen of lifelong aspirin and warfarin for 45 days (n = 26, 47%) or lifelong aspirin and a direct oral anticoagulant (apixaban, rivaroxaban, or dabigatran) for 45 days (n = 17, 31%). A minority of patients were treated with a combination of aspirin and clopidogrel (n = 9, 16%), whereas 2 patients were treated with direct oral anticoagulant monotherapy and 1 was treated with warfarin alone. Forty-five days postimplant, patients were transitioned to lifelong aspirin monotherapy.

The median follow-up for the study cohort was 1.6 years (Q1-Q3: 1.1-2.6 years). Over the first year, ischemic stroke occurred in 1 patient (1.4%), and bleeding complications occurred in 5 (10.7%) patients. The cumulative incidence of ischemic stroke was 1.4% at 1 year and 3.4% at 5 years. Neither of the patients who suffered a post–device implantation ischemic stroke had a prior history of stroke. Among the patients who suffered a significant bleeding event during follow-up, 4 had a history of a major bleeding event before device implantation; 1 had a hematologic malignancy (multiple myeloma). One year device-related complications based on the cumulative incidence occurred in 3 (5.6%) patients: 2 (4.2%) patients developed device thrombosis, and 1 patient (1.1%) developed a peridevice leak >5 mm. An additional patient developed a peridevice leak between 1 and 5 years. At 5 years post–LAAO device implantation, 7.2% experienced device thrombosis; this was observed in 4 patients at 1, 10, 10, and 23 months postimplant. All of them were on aspirin at the time of thrombosis; 2 patients had active malignancy (squamous cell carcinoma of the lung and metastatic hepatocellular carcinoma), and 2 were in remission. All were treated with 3 to 6 months of oral anticoagulation with resolution of thrombus. Overall, 1-year mortality based on the cumulative incidence function was 6.5% (n = 3) with 14 deaths at the last follow-up and the most common causes being malignancy progression (n = 5) and infection (n = 4). The fatal infections were caused by sepsis from cholecystitis, osteomyelitis, urosepsis, and prosthetic joint infection, all unrelated to the LAAO.

**Figure 1:**
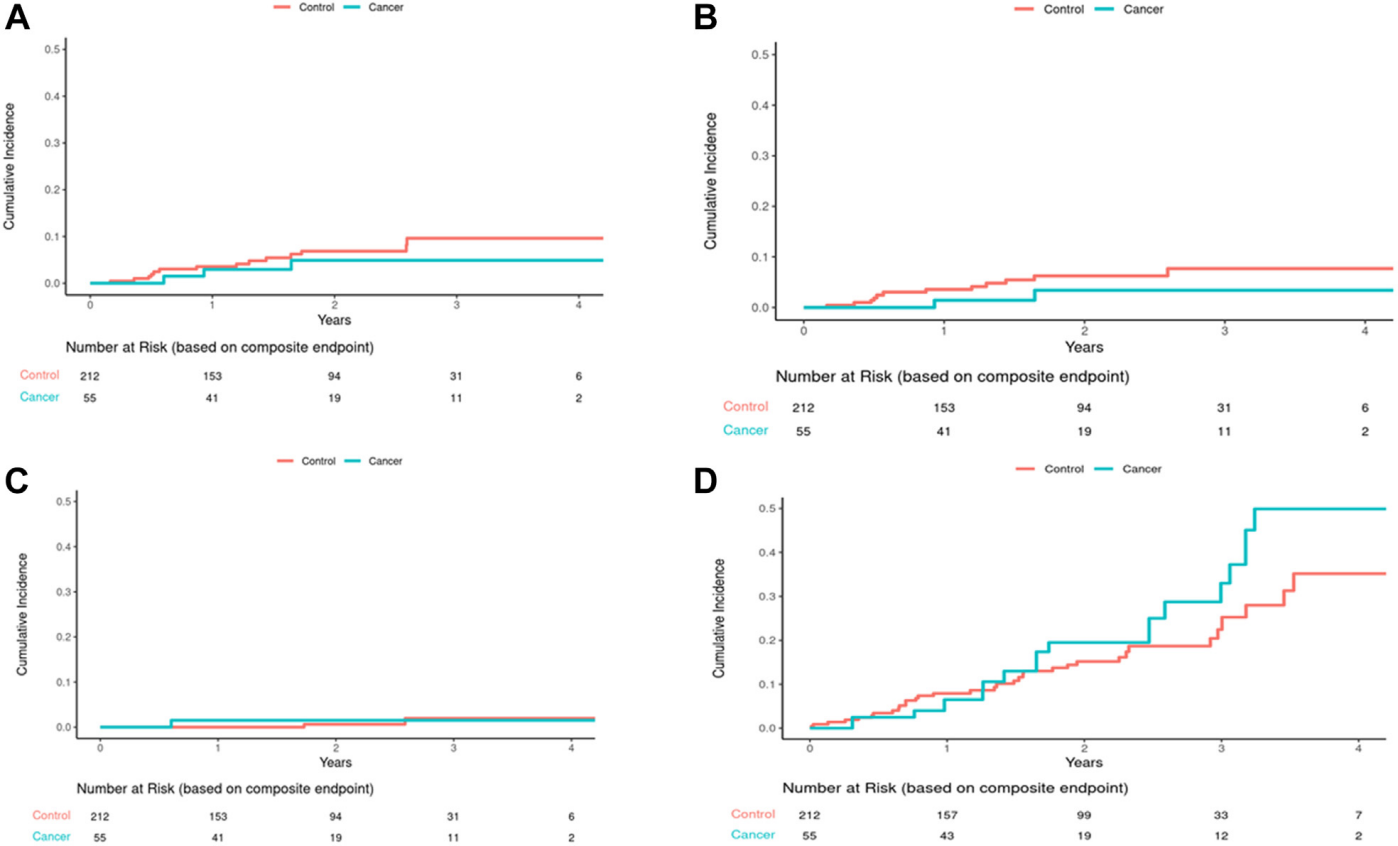
Cumulative Incidence for Stroke and Death (Cancer vs Noncancer Patients) **(A)** No statistical difference in the rate of combined stroke between cancer and noncancer patients (cause-specific HR: 0.60; 95% CI: 0.17-2.10; *P =* 0.43; subdistribution HR: 0.59; 95% CI: 0.17-2.07; *P =* 0.41). **(B)** No statistical difference in the rate of ischemic stroke between cancer and noncancer patients (cause-specific HR: 0.44; 95% CI: 0.10-1.97; *P =* 0.28; subdistribution HR: 0.43; 95% CI: 0.10-1.93; *P =* 0.27). **(C)** No statistical difference in the rate of hemorrhagic stroke between cancer and non-cancer patients (cause-specific HR: 1.71; 95% CI: 0.17-17.3; *P =* 0.65; subdistribution HR: 1.70; 95% CI: 0.16-17.89; *P =* 0.66). **(D)** No statistical difference in the rate of death between cancer and noncancer patients (HR: 1.39; 95% CI: 0.73-2.64; *P =* 0.32).

**Figure 2:**
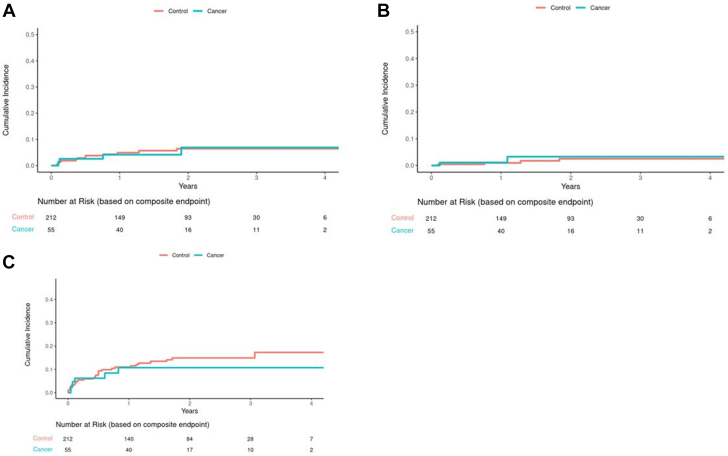
Cumulative Incidence for Device and Bleeding Complications (Cancer vs Noncancer Patients) **(A)** No statistical difference in the rate of device thrombosis between cancer and noncancer patients (cause-specific HR: 1.65; 95% CI: 0.48-5.69; *P =* 0.43; subdistribution HR: 1.65; 95% CI: 0.48-5.68; *P =* 0.42). **(B)** No statistical difference in the rate of device perileak between cancer and noncancer patients (cause-specific HR: 1.52; 95% CI: 0.27-8.49; *P =* 0.63; subdistribution HR: 1.50; 95% CI: 0.27-8.32; *P =* 0.64). **(C)** No statistical difference in the rate of bleeding complications between cancer and noncancer patients (cause-specific HR: 0.72; 95% CI: 0.28-1.86; *P =* 0.49; subdistribution HR: 0.73; 95% CI: 0.28-1.89; *P =* 0.52).

**Figure 3:**
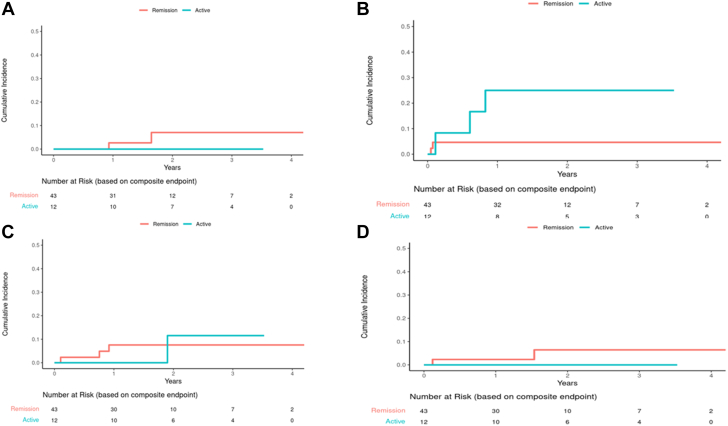
Cumulative Incidence for Clinical Outcomes (Active Cancer vs Remission) **(A)** No statistical difference in the rate of ischemic stroke between active cancer patients versus remission (*P =* 0.99). **(B)** No statistical difference in bleeding complications between active cancer patients vs remission (*P =* 0.07). **(C)** No statistical difference in the rate of device thrombosis between active cancer patients vs remission (*P =* 0.92). **(D)** No statistical difference in the rate of device perileak between active cancer patients vs remission (*P =* 0.99).

Compared with matched controls who underwent LAAO without evidence of malignancy (n = 212) with a median follow-up of 1.9 years (Q1-Q3: 0.9-2.6 years) (Table 1), patients with cancer had no significant difference in mortality (HR: 1.39; 95% CI: 0.73-2.64; *P =* 0.32). However, an increase in mortality in patients with cancer became noticeable in Kaplan-Meier curves toward the end of the study period (Figure 1). Similarly, there was no difference in the incidence of stroke (cause-specific HR: 0.60; 95% CI: 0.17-2.10; *P =* 0.43) or bleeding events (cause-specific HR: 0.72; 95% CI: 0.28-1.86; *P =* 0.49) between patients with malignancy and those without. There was also no difference in the rate of device thrombosis (cause-specific HR: 1.65; 95% CI: 0.48-5.69; *P =* 0.43) or perileak (cause-specific HR: 1.52; 95% CI: 0.27-8.49; *P =* 0.63) between patients with malignancy and those without (Figure 2). Sensitivity analysis using subdistribution hazards yielded similar results. When patients with active malignancy (n = 12) were compared with those in remission (n = 43), there was no significant difference in ischemic stroke (*P =* 0.99), bleeding complications (*P =* 0.069), device thrombosis (*P =* 0.92), or device perileak (*P =* 0.99) (Figure 3). Although there was a slightly higher cumulative incidence of bleeding events in patients with active cancer (Figure 3B), the sample size was small, and the incidence did not meet statistical significance.

## Discussion

To our knowledge, this is the first report on the procedural success and outcomes of LAAO device implantation among patients with AF and cancer (Central Illustration). Salient findings include the following: 1) patients were at high risk for both stroke (median CHA_2_Ds_2_-VASc score 5) and bleeding (85.5% with a prior bleeding event); 2) acute procedural success was achieved in 100%; 3) during the first year, the cumulative incidence estimates of device-related complications, ischemic stroke, and bleeding were noted as 4.2%, 1.4%, and 10.7%, respectively; and 4) there were no significant differences in these outcomes compared with patients without a history of malignancy.

**Central Illustration:**
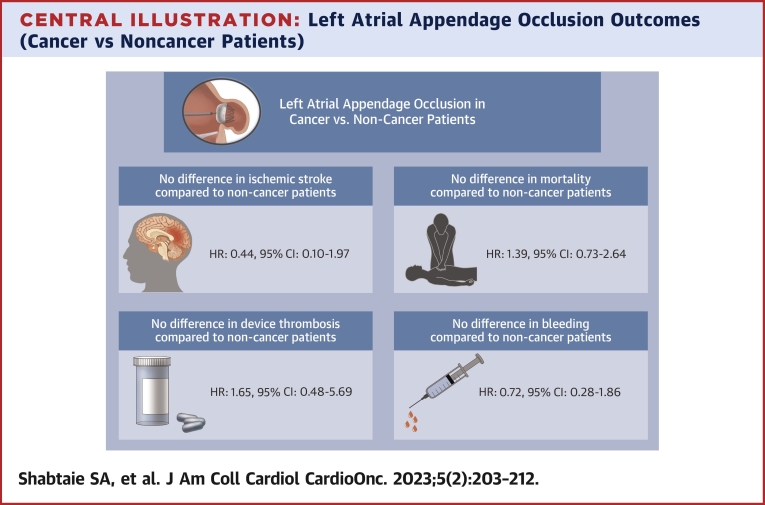
Left Atrial Appendage Occlusion Outcomes (Cancer vs Noncancer Patients) A summary of left atrial appendage occlusion (LAAO) outcomes in atrial fibrillation patients with and without cancer. Overall, LAAO offered a reduction in stroke at no increased bleeding risk similar to noncancer patients.

Our results provide evidence that LAAO is a reasonable consideration for stroke prophylaxis in patients with a background history of malignancy (both active and in remission). After the Watchman device received Food and Drug Administration approval in 2015, there has been an increasing amount of information available regarding the efficacy and safety profile of LAAO device implantation in “real-world” clinical practice.^18^ Such data are critical given the strict recruitment processes for the pivotal PROTECT AF (Percutaneous Closure of the Left Atrial Appendage Versus Warfarin Therapy for Prevention of Stroke in Patients With Atrial Fibrillation) and PREVAIL (Evaluation of the WATCHMAN LAA Closure Device in Patients With Atrial Fibrillation Versus Long Term Warfarin Therapy) trials.^19^ Notably, patients with a history of cancer were not specifically included or even described in these trials, making it challenging to ascertain the utility of LAAO device implantation in this population. This is especially important to address because cancer patients are intrinsically at increased risk for both thromboembolism as well as bleeding, either with or without anticoagulants.^9^^,^^11^^,^^13^^,^^20^ Not all cancers carry the same thrombotic risk; breast and prostate cancers are associated with lower risk, whereas hematologic, pancreatic, lung, stomach, and brain cancers portend a much higher risk.^21^ Furthermore, the presence of metastatic disease, surgery, and the type of chemotherapeutic and the type of cancer therapeutic impact risk.^22^ Within our cohort, the most prevalent malignancy was prostate cancer (29.1%), although a large diversity of malignancies and treatments was represented.

Indeed, the median CHA_2_Ds_2_-VASc scores from our study cohort,^5^ driven in large part by more advanced age alongside prior stroke/transient ischemic attack, were far higher than the mean observed in the PREVAIL trial (3.8), although it was comparable to what was observed in the National Cardiovascular Data Registry (4.6).^6^^,^^23^ Additionally, most patients had experienced a significant bleed before with intracranial and gastrointestinal etiologies constituting the majority of events, and 89.1% of patients were on anticoagulation antecedent to LAAO. Thus, there is a delicate balance between stroke and bleeding risk in many patients with AF and cancer, highlighting the clinical rationale for considering LAAO device implantation in this high-risk group.

Acute procedural success was achieved in all patients in the present study. This was not unanticipated in a high-volume center and commensurate with contemporary registry data.^7^^,^^23^ Although 6 (10.9%) complications within a 30-day window were noted, two-thirds of these events were groin hematomas, which were self-limiting and did not require surgical repair. Hence, periprocedural results were quite reasonable in this patient population.

With regard to device-related complications, 4 patients were noted to have device thrombus, and 2 experienced a >5-mm peridevice leak. The former is of particular concern because its presence has been associated with an elevated risk of ischemic stroke in prior studies.^24^^,^^25^ Data regarding the management of device thrombus are limited, although 1 small study showed successful thrombus resolution with a 6-week course of warfarin.^26^ In our study, anticoagulation was resumed for a short time in all 4 patients, with no consequent stroke or bleeding episodes noted thereafter. The clinical implications of peridevice leaks remain unclear because studies have not consistently shown a significant association with adverse events.^27^^,^^28^ Approaches for perileak closures have been developed, although the indications and timing for these techniques are also not well-defined. The patients with a peridevice leak in this study were managed conservatively with no clinical sequelae observed. Given the limited number of events, further studies regarding the optimal surveillance and management of device-related complications in this patient population will be needed. It is noted that many LAAO device implantation adverse events, such as device thrombosis, occur >1 month postimplantation, as seen in our cohort, and further long-term follow-up is prudent to define the true prevalence of events.^29^

Three patients experienced strokes over the follow-up period (2 ischemic and 1 hemorrhagic), and 5 patients experienced clinically significant bleeding; however, 3 of these occurred during the 45-day postimplantation window (while they were on anticoagulation/antiplatelet therapy). These observations highlight the challenges faced with using anticoagulants in this patient population as well as the reassuring efficacy for stroke prevention despite the elevated stroke risk based on CHA_2_Ds_2_-VASc scores. Over one-quarter of patients died during the follow-up period, primarily from their underlying malignancy or severe infection. Again, this was not surprising given the study cohort’s inherent comorbidities. Importantly, no patients were deemed to experience mortality or infection related to the LAAO device. Finally, when compared with an inverse probability–weighted control cohort without cancer, there were no statistically significant differences in the previously mentioned outcomes. Therefore, these analyses suggest that LAAO device implantation may be an effective and safe strategy for patients with AF and cancer.

### Study limitations

This was a retrospective cohort study of a high-volume referral health system. The follow-up period was relatively short, which precludes conclusions regarding long-term outcomes in this patient population. The follow-up duration was limited because the majority of LAAO devices were implanted in 2018 and 2019. Furthermore, there was a significant amount of heterogeneity with regard to patient malignancy type and staging, not allowing though for robust subgroup analyses. For instance, given the limitations of our study's cohort size, there was a small group with hematologic malignancy, which may have higher rates of blood dyscrasias and hence higher bleeding rates. The minority of patients (21.8%) had active malignancy at the time of device implantation. Furthermore, most of the patients studied in our cohort were elderly (mean age 79.0 ± 6.1 years), so the results may not be generalizable to younger patients. Finally, no patients received the newer-generation Watchman FLX device (Boston Scientific) or the recently approved Amplatz Amulet device (Abbott). Given the limited data available on LAAO in patients with malignancy, this does contribute to the limited literature on the topic.

## Conclusions

Within our cohort, LAAO in patients with AF and cancer offered reasonable acute procedural success with a low incidence of ischemic stroke similar to that of patients without malignancy. Further multicenter studies are needed to assess LAAO in patients with active malignancy with anticipated blood dyscrasias.

Perspectives **COMPETENCY IN MEDICAL KNOWLEDGE:** Patients with cancer and atrial fibrillation are at increased bleeding and stroke risk; yet, oral anticoagulation portends an increased risk of bleeding compared with the general population. In our cohort derived from a tertiary academic center, left atrial appendage occlusion offered a reasonable reduction in stroke with no excess bleeding risk similar to patients without cancer.

**TRANSLATIONAL OUTLOOK:** Larger, multicenter, and adequately powered studies are needed to assess left atrial appendage occlusion efficacy at reducing stroke in cancer patients with a focus on those with active cancer, hematologic malignancies, younger patients with cancer, and novel left atrial appendage occlusion devices.

## Funding Support and Author Disclosures

This study was funded by the National Institutes of Health/National Cancer Institute (R01CA233601) and the Miami Heart Foundation. Dr Holmes is a member of the advisory board (unpaid) for Boston Scientific. Dr Herrmann is on the advisory board (unrelated to the topic of this study) for Pfizer. All other authors have reported that they have no relationships relevant to the contents of this paper to disclose.
